# Cross-Linking of Polypropylene with Thiophene and Imidazole

**DOI:** 10.3390/polym14112198

**Published:** 2022-05-28

**Authors:** Henky Muljana, Klaas Remerie, Gert Boven, Francesco Picchioni, Ranjita K. Bose

**Affiliations:** 1 Department of Chemical Engineering, Parahyangan Catholic University, Ciumbuleuit 94, Bandung 40141, Indonesia; 2 Department of Chemical Engineering, ENTEG, University of Groningen, Nijenborgh 4, 9747 AG Groningen, The Netherlands; f.picchioni@rug.nl; 3 SABIC, Plasticslaan 1, P.O. Box 117, 4600 AC Bergen op Zoom, The Netherlands; k.remerie@home.nl (K.R.); gert.boven@sabic.com (G.B.)

**Keywords:** polypropylene, cross-linked, Diels–Alder, thiophene-bismaleimide, imidazole-bismaleimide, thermo-reversible

## Abstract

In this work, two novel routes to synthesis cross-linked polypropylene (PP) are introduced by using two different precursors (2-thiophenemethyl amine (TMA) and 1-(3 aminopropyl) imidazole (API)), both cross-linked with 1,1′-(methylenedi-4,1-phenylene) bismaleimide (BM) at two different annealing temperature values (*T* = 50 °C and *T* = 150 °C). Both Diels–Alder (DA) and Michael addition reactions were successfully performed with TMA and API, respectively, albeit with different reactivity. Imidazole clearly shows a higher reactivity compared to thiophene. In addition, an increase in annealing temperature leads to a higher degree of cross-linking. The highest degree of cross-linking was obtained by the imidazole product after annealing at 150 °C (IMG1A150) as evident from the highest complex viscosity (|η*|) value of IMG1A150. A difference in rheology and thermal properties between the imidazole and thiophene cross-linked products was also observed. However, both products have superior melt properties and thermal stability compared with the starting material. They show processability at high temperatures. The melt flow behavior and de-cross-linking at higher temperatures can be tuned depending on the choice of imidazole or thiophene. This study shows an advance on the cross-linked PP processing and its product performances for further application on the commercial scale.

## 1. Introduction

Cross-linking reaction is carried out to improve physical and chemical properties of the polymer/biopolymer and expand the applicability of the cross-linked materials in various applications, such as in food and pharmaceutical, biomedical, electronics and packaging [1,2,3,4,5,6]. The synthesis of cross-linked polypropylene (PP) has gained significant attention from many researchers and has become one of the crucial research topics in the field of PP modification. Even though neat PP has excellent properties such as high toughness, high impact and tensile strength and strong resistance to heat and chemicals [7,8,9,10,11], cross-linked PP improves upon some drawbacks in neat PP properties [12,13]. After cross-linking, the modified PP product is expected to gain a higher thermal stability, an increase in electrical discharge and chemical resistance, higher melt strength and an improvement in the resistance towards creep and stress cracking [1,12,14]. The cross-linked PP is suitable to be used as a cable insulation (gel content of 55%), an electric energy storage (capacitor), highly foamed materials and a compatibilizer in blends containing PP [2,15]. Conventional cross-linking of PP proceeds via macro radical formation through several possible routes such as thermal decomposition using peroxides, high energy irradiation (gamma and electron beams), ultraviolet (UV) radiation and silane grafting with moisture cross-linking [12,14,15,16]. However, the main disadvantage of the current process is the possible degradation (β scission) in the PP backbone and this limits further commercialization of the process on the larger scale [12,14].

Another possible route to synthesize cross-linked PP is using Diels–Alder (DA) chemistry. This chemistry has been widely introduced in the cross-linking reaction of many polymer systems due to its fast kinetics and more importantly due to the requirement of relatively mild reaction conditions [17]. An in-depth study on the Diels–Alder in polymer chemistry and its potential future application has also been discussed in the recent publication [18]. In this respect, the application of DA in the cross-linking of PP is beneficial to reduce the degradation in the PP backbone. DA involves a diene (electron rich) and a dienophile (electron poor) in the reaction [17]. Among others, a furan and a bismaleimide (BM) is widely used as the pair of diene and dienophile in DA cross-linking of various polymer systems, for instance in polyketones [19,20,21], polymethacrylate containing furan and maleimide functionalities [22], polyurethane [23], maleated ethylene propylene rubber (EPM) [17,24], polyfurfural alcohol [25] and Lotader^®^ (polyethylene-co-glycidyl methacrylate) [26].

Furthermore, we recently reported the successful application of furan—BM as the cross-linking agent using maleated PP (PPgMA) as the starting material. In the report, we demonstrated a significant influence of maleic anhydride (MA) content and annealing temperature on the degree of cross-linking of the final products [27]. We also noticed that the furan grafting percentage on the maleated PP is relatively low (% grafted = 26%). This may affect the reactivity of the second reaction step (DA with BM) and eventually the properties of the final products. Therefore, further studies are required to improve the grafting efficiency in the reaction.

**Scheme 1:**
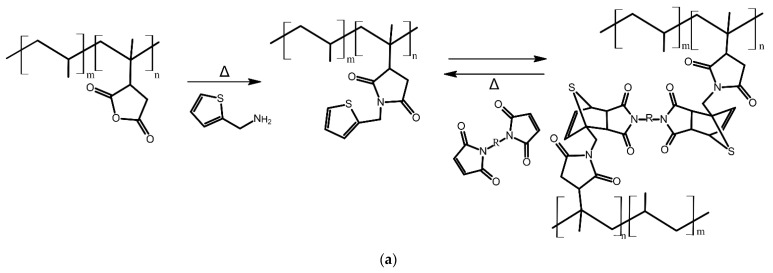

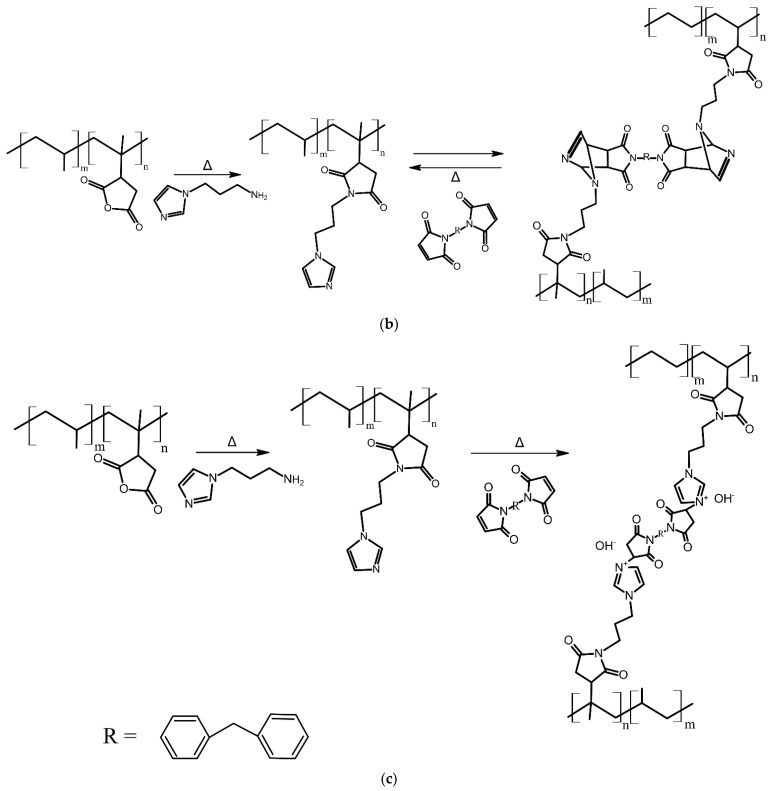
The proposed grafting reaction of thiophene (**a**), imidazole (**b**) followed by Diels–Alder (DA) cross-linking with bismaleimide and (**c**) Michael addition between imidazole and bismaleimide.

There are two approaches that can be used to improve the grafting efficiency, the first approach is conducting systematic studies to optimize the important process variables in the reaction and the second approach is exploring another potential diene precursor instead of furfuryl amine (FFA) as the cross-linking agent. In this paper, we focus on the latter approach while the optimization study will be reported in our next publication. Among aromatic amines with five heterocycle compounds, 2-thiophenemethyl amine (TMA) and 1-(3 aminopropyl) imidazole (API) have the potential to be used as a diene precursor as well (Scheme 1a,b). To the best of our knowledge, there is not so much open literature available regarding the application of thiophene or imidazole in Diels–Alder of polymer cross-linking reaction. Only the work of Polgar and coworkers reported the successful application of TMA in the functionalization of maleated ethylene propylene rubber (EPM) and cross-linking with bismaleimide (BM) [28] while no information is available in literature on the application of imidazole and BM as the pair of diene and dienophile in the cross-linking of polymer systems. This gives a strong incentive to investigate the potential application of the two reagents in the cross-linking reaction of PP.

In addition to the latter, it was reported in the literature that imidazole ring and bismaleimide can react via Michael addition chemistry in which imidazole and bismaleimide act as a pair of Michael donor and acceptor, respectively [29]. In the conjugative addition reaction, imidazole containing nitrogen nucleophile is reacted with unsaturated carbon-carbon of bismaleimide to produce a new saturated carbon-carbon bond (Scheme 1c) [29]. In this respect, not only via DA reaction route, the cross-linking reaction involving imidazole and bismaleimide is also possible to proceed via Michael addition chemistry (Scheme 1c) resulting with different product properties between the two synthetic pathways (i.e., reversibility of the cross-linked product). These properties can be beneficial for further application of these products on the commercial scale.

The aim of this work is to investigate the application of TMA and API as a novel precursor in the synthesis of cross-linked PP. In this work, we limit our research scope by using commercially available maleated PP since the synthesis of this material has been extensively explored and reported in open literature [30,31,32,33]. The reaction proceeds via grafting of TMA or API onto maleated PP and follows with the DA reaction between pendant thiophene and BM (see Scheme 1a), while for API, the imidazole pendant cross-linked reaction with BM can proceed via DA or Michael addition (Scheme 1b,c).

This study not only focuses on evaluating different reactivities between TMA and API on both reaction steps (grafting of maleated PP and cross-linking with BM) but also to investigate possible cross-linking reaction pathways between DA or Michael addition that proceed between API and BM. In addition, the different annealing temperatures and the influence of both TMA and API moieties on the properties of final products were also investigated. The relevant product properties were determined using Fourier transform infrared (FT-IR) spectroscopy, oscillatory shear rheology, solubility measurements, differential scanning calorimetry (DSC) and thermogravimetric analysis (TGA).

## 2. Materials and Methods

### 2.1. Materials

Polypropylene grafted maleic anhydride (PPgMA, average Mn 3900 g/mol, average Mw 9100 g/mol and 8–10% wt. maleic anhydride) was purchased from Sigma Aldrich, Munich, Germany. Analytical grade 2-thiophenemethylamine (TMA, 96%), 1-(3-aminopropyl)imidazole (API, >97%), 1,1′-(methylenedi-4,1-phenylene) bismaleimide (BM, 95%), tetrahydrofuran (THF, >99.9%), chloroform (CHCl_3_, >99%) were purchased from Sigma Aldrich, Munich, Germany. Analytical grade 1-2 dichlorobenzene (DCB, >99%) was purchased from Fluka, Landsmeer, the Netherlands. Antioxidant AOB225 (CAS Number 9421-57-8) was kindly supplied by SABIC, Geleen, the Netherlands. All chemicals were directly used without any further purification.

### 2.2. Experimental Procedures

#### 2.2.1. Functionalization with Different Aromatic Amines (Thiophene and Imidazole) on PPgMA

PPgMA (15 g) and 4 equivalents (based on MA content) of TMA/API were added in a Brabender kneader and mixed for 10 min at temperature of 160 °C and rotational speed of 50 rpm. A slightly different product works up the procedure between TMA and API functionalized products was applied. The unreacted TMA was extracted from the grinded products at 170 °C for 3 h using THF as the solvent in a SOXTEC apparatus. The solid products were dried in a vacuum oven at 50 °C until no further mass loss was observed. The final step in the product work up involves a compression of the dried product for 30 min at a temperature of 175 °C and pressure of 100 bars to close the amine aromatic ring and complete the reaction [17].

For API functionalized product, after reaction in the Brabender, the product was washed in 250 mL of water, stirred for 1.5 h and filtrated. The washing procedure was repeated twice to ensure the removal of unreacted imidazole from the products. Next, the products were dried in a vacuum oven at 70 °C until they had a constant weight and compressed with the same condition as described above.

#### 2.2.2. Cross-Linking of Functionalized PPgMA with Bismaleimide

The thiophene/imidazole grafted product (2 g), antioxidants (4 mg), 0.5 equivalent of BM based on the theoretical amount of grafted thiophene/imidazole in the product and CHCl_3_ (20 gr) as the solvent were charged into 25 mL of round bottom flask. The reaction was allowed to proceed at 50 °C for 3 h. Afterwards, the mixture was collected in a glass beaker and stored in the fume cabinet for 24 h to allow the complete removal of CHCl_3_ from the products by slow evaporation. Finally, the annealing step at different temperatures (50–150 °C) for 24 h was carried out on the solid products.

#### 2.2.3. Solubility Test of the Cross-Linked Products

The differences in the extent of cross-linking and de-cross-linking of the annealed product for both TMA and API were qualitatively determined using a solubility test for which DCB was chosen as the solvent. The solid annealed product of TMA or API (0.05 mg) were added into a 5 mL testing bottle. Subsequently, dichlorobenzene (DCB, 2g) was charged into the testing bottle. The mixture was heated up in an oven at 120 °C for 24 h. Solubility of the annealed product was then observed by comparing the photograph of all product that was taken before (t = 0) and after heating up in the oven for 24 h (t = 24 h).

### 2.3. Analytical Equipment

The FT-IR analysis was performed using a Shimadzu IRTracer-100 (Shimadzu, Kyoto, Japan). The FT-IR spectra were taken on an attenuated total reflectance (ATR) Golden Gate Specac Golden Gate ATR Top and West 6100+ temperature controller (Specac, Philadelphia, PA, USA) in the absorption range of 500–4000 cm^−1^ with 64 scans and a resolution of 4 cm^−1^. The quantification of the intensity of the relevant peaks was done after deconvolution (R^2^ > 0.95).

The thermal properties of the products were analyzed on a Perkin Elmer TGA 4000 (Perkin Elmer, Waltham, MA, USA) and a Perkin Elmer DSC 7 (Perkin Elmer, Shelton, ST, USA). TGA analysis were performed within a temperature range of 30–700 °C (heating rate 10 °C/min) in an inert atmosphere. DSC thermograms were acquired in two heating and cooling cycles, where the first cycle is necessary to remove thermal history from the sample. The heating step was done in the temperature range of 25–200 °C with a heating rate of 5 °C min^−1^ while, in the cooling step, the sample was cooled back to 25 °C with a cooling rate of 5 °C min^−1^.

The composition of C, H, N and S in the product were analyzed using a Euro EA 3000 Eurovector S.P.A Elemental Analyzer (Eurovector, Langenselbold, Germany).

The rheology of the products was analyzed in a controlled test chamber (CTC) Haake Mars III (Thermo Fisher Scientific, Karlsruhe, Germany). Prior to the measurement, a sample disc (diameter of 2.5 cm and thickness of 1 mm) was prepared in a compression molding (Taunus Ton press type VS) at temperature of 155 °C and pressure of 100 bars for 10 min. Next, the press was cooled down to room temperature using cooling water for approximately 20 min.

All of the rheology measurements were performed in the linear viscoelastic regime (strain of 1%). Temperature sweep experiment was done in the temperature range of 150–200 °C (5 °C/min) at an angular frequency (ω) of 1 rad/s. Frequency sweep experiments were performed in the frequency range of 0.01–100 rad/s and a temperature of 160 °C. 

The melt volume rate (MVR) of the products was measured using an MVR instrument Zwick/Roell BMF 001 (Zwick/Roell, Ulm, Germany) at a temperature range of 170–230 °C under loading of 2.16 kg and 5 kg.

## 3. Results and Discussion

The cross-linking reactions of maleated PP (PPgMA) were done by first functionalizing PP with two different reactants (thiophene and imidazole) and secondly cross-linking them with 1,1′-(methylenedi-4,1-phenylene) bismaleimide (BM) at two annealing temperatures. A number of experiments were performed according to the reaction Scheme 1 (see Table 1).

**Figure 1:**
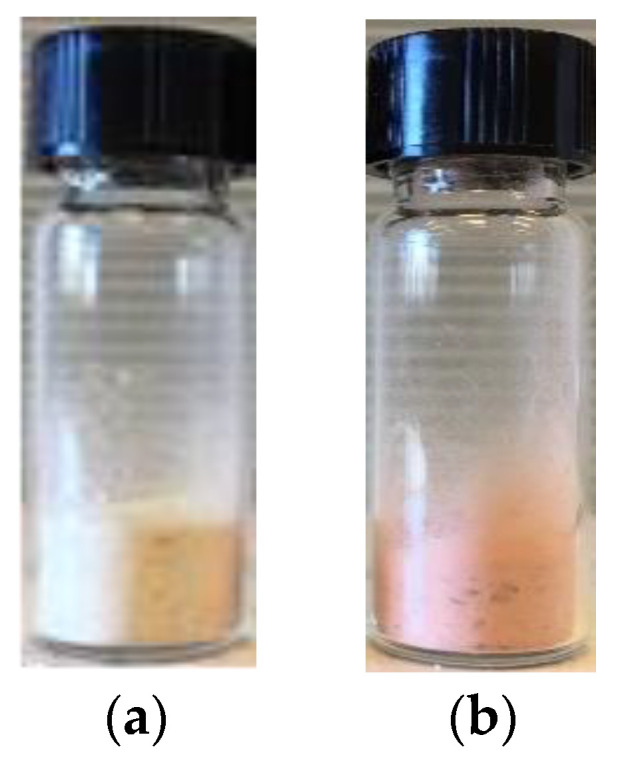
Photographs of the products after cross-linking reaction between thiophene grafted (**a**) and imidazole grafted; (**b**) products with BM.

Interestingly, differences in the product appearances between thiophene (TG1) and imidazole (IMG1) cross-linked products were observed. TG1 has a slight yellow color while IMG1 has a pink color (Figure 1). Despite the color differences, the differences in reactivity and the product properties between the two compounds were also observed. More detailed explanation for each reaction step (Scheme 1) including the proof of principles and relevant product properties are discussed in detail in the following section.

### 3.1. Grafting of Thiophene and Imidazole onto Maleated PP

**Figure 2:**
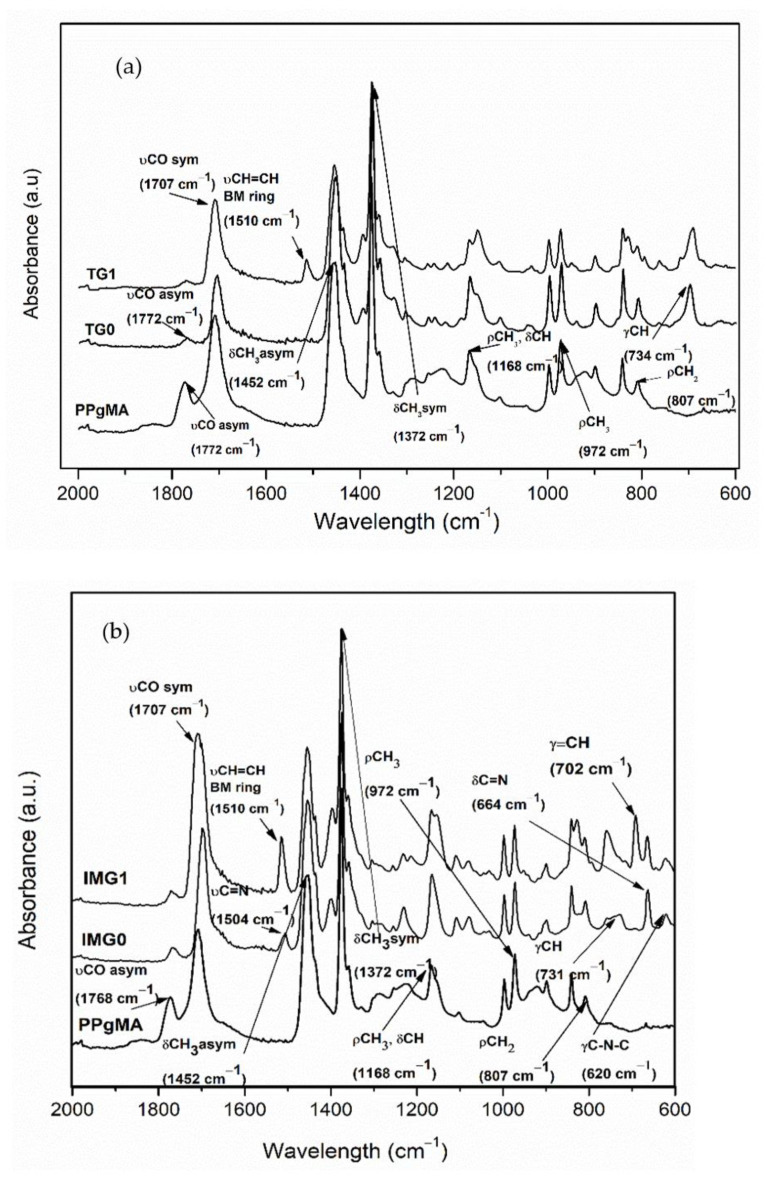
FTIR spectra of maleated PP and its derivatives with thiophene (**a**) and imidazole (**b**) in the absorption range of 600−2000 cm^−1^.

The change in the chemical structure of PPgMA after grafting reaction was followed by FT-IR. The FT-IR spectra of thiophene (TG0) and imidazole (IMG0) grafted products are given in Figure 2a,b, respectively. When comparing the spectra of TG0 with the starting material (Figure 1a), it is obvious that both spectra show several strong absorption bands at 1452–1453 cm^−1^ (CH_3_ asymmetric bending, δCH_3_ asym), 1370–1375 cm^−1^ (CH_3_ symmetric bending, δCH_3_ sym), 1165–1168 cm^−1^ (CH_3_ rocking, ρCH_3_ and CH bending, δCH), 970–972 cm^−1^ (CH_3_ rocking ρCH_3_) and 807–810 cm^−1^ (CH_2_ rocking, ρCH_2_) [34,35]. These peaks belong to the chemical group existed in the neat PP. In addition, the typical peaks for the anhydride group in PPgMA appear in the absorption band of 1768–1772 cm^−1^ (C=O asymmetric stretching, υCOasym) and 1710–1715 cm^−1^ (C=O symmetric stretching, υCOsym) [17,30,34,36].

It is evident that the intensity of the peak at 1768–1772 cm^−1^ (C=O asymmetric stretching, υCOasym) decreases and, more importantly, a new peak at absorption band 700 cm^−1^ appears after modification [37,38,39]. The new peak corresponds to the CH out of plane bending vibrations of (γCH) of the thiophene ring (Figure 2a). Moreover, after grafting with imidazole (Figure 2b), the presence of imidazole pendant group in the PP backbone is indicated by the peaks at 620–622 cm^−1^ (out of plane C–N–C bending of imidazole rings, γC–N–C), at 664–665 cm^−1^ (C=N bending of imidazole rings, δC=N), at 731–735 cm^−1^ (out of plane CH bending of imidazole rings, γCH) and at 1504–1510 cm^−1^ (imidazole ring stretching, υ imidazole ring and C=N stretching of imidazole rings, υC=N) [37,40,41]. The decrease in the intensity of anhydride peak (at 1768–1772 cm^−1^) was also observed in IMG0 spectra (Figure 2b). These observations imply that the functionalization of PPgMA with both thiophene and imidazole were successful albeit with different reactivity.

The difference in grafting efficiency of thiophene and imidazole pendant in the products is quantified by elemental analysis (Table 2). The calculation is made based on the ratio between the nitrogen content (presence in TG0 and IMG0) with the total amount of maleic anhydride (MA) in the PPgMA. It is apparent that the percentage of grafted IMG0 (65% wt.) is higher compared with TG0 (29.8% wt.) and also with furan grafted product (26.3% wt.) [27]. Clearly, API is far more reactive compared with TMA and furfuryl amine (FFA). 

**Scheme 2:**
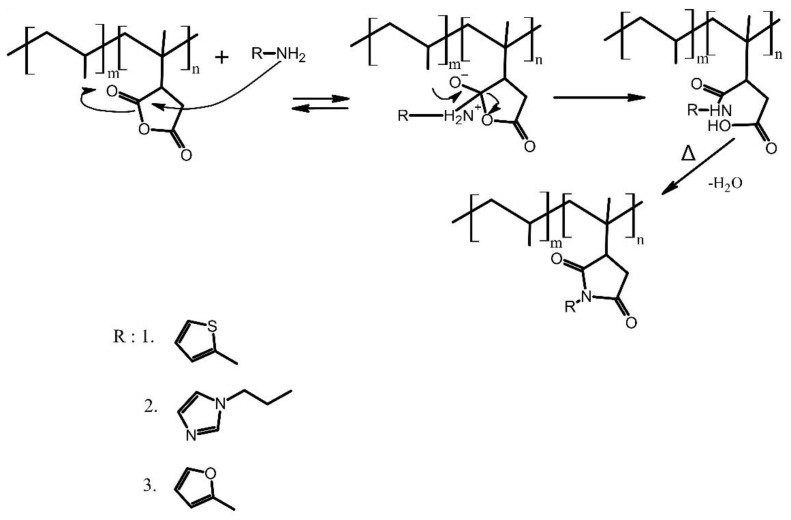
The proposed grafting reaction mechanism of maleated polypropylene (PPgMA) with different aromatic amine (thiophenemethyl amine (1), aminopropyl imidazole (2) and furfuryl amine (3)).

The grafting reaction is likely to proceed via nucleophilic substitution mechanism (Scheme 2). Here, TMA, FFA, or API is a nucleophile while the anhydride is an electrophile. The grafting reaction starts with the reaction between the amine functional group in TMA, FFA or API with the carbonyl group of the anhydride, opening the cyclic rings and forming the intermediate, an amide carboxylic acid (Scheme 2). The reaction is completed with the closure of the ring of the intermediate amide carboxylic acid at high temperature and formed the thiophene or imidazole grafted products [17,42]. 

Based on this mechanism, it is reasonable to assume that the difference in the reactivity of the nucleophilic substitution step (Scheme 2) depends on the different in the nucleophilicity of the amine functional group between TMA and API. Aminopropyl imidazole (API) with the longer alkyl chain (–C_3_H_6_) has a stronger inductive donating effect on the amine group compared with thiophene methyl amine (TMA) and Furfuryl Amine (FFA) with a shorter alkyl chain (–CH_2_) [43]. This leads to a higher nucleophilicity of the amines group in API and eventually enhances its reactivity in the grafting reaction (Table 2).

### 3.2. Cross-Linking of Thiophene and Imidazole Moieties with BM

Figure 2 shows the changes in the FT-IR spectra of thiophene (Figure 2a) and imidazole (Figure 2b) grafted products after cross-linked reaction with BM. Compared with the spectra of thiophene grafted product (TG0), after cross-linking, an additional peak at 1510–1513 cm^−1^ was present in the TG1 spectra. This peak is assigned to CH=CH of the BM rings stretching vibrations (υCH=CH) [44,45]. The same peak (υCH=CH) appeared in the spectrum of cross-linked grafted imidazole products (IMG1) at 1510 cm^−1^ (Figure 2b), overlapping with the peak of imidazole ring stretching (υ imidazole ring) [40,44]. As can be seen in Figure 2b, an additional peak at 702 cm^−1^ which is assigned to =CH out of plane bending vibration of the maleimide ring (γ = CH), appears in the spectra of IMG1 [46,47].

**Figure 3:**
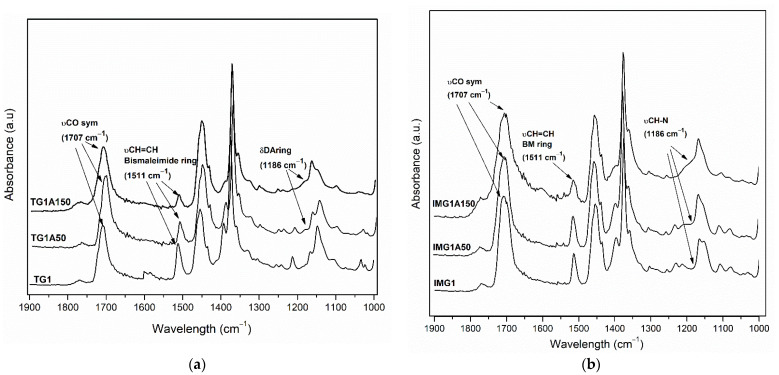
FTIR spectra of the products after Diels–Alder reaction with thiophene (**a**) and imidazole (**b**) and annealing with different temperatures in the absorption range of 1000−1900 cm^−1^.

Another important peak to confirm the presence of cross-linked adduct is the peak at 1186 cm^−1^ which corresponds to the cyclo DA adduct [17,19,48]. Moreover, the same peak can also be assigned to CH–N aliphatic stretching (υCH–N) if the cross-linked proceeds via Michael addition reaction (Scheme 1c) [49,50,51]. As clearly shown in Figure 3a, a clear shoulder at 1186 cm^−1^ appears after annealing, especially at a temperature of 150 °C for both TG1 (Figure 3a) and IMG1 (Figure 3b). This shoulder may be strongly related with the appearance of the DA adduct for TG1 and its annealed product. Although a shoulder at absorption band of 1186 cm^−1^ also appears in the spectra of IMG1 and its annealed product, it is still uncertain whether the cross-linking between imidazole and bismaleimide proceeds via Diels–Alder or Michael addition pathways. The thermal reversibility analysis of the final product using the rheology measurements (see Section 3.3) can be used to elucidate the possible cross-linking reaction pathways between imidazole and bismaleimide.

In addition to the confirmation regarding the successful cross-linking reaction, Figure 3 also shows an increase in cross-linking at higher annealing temperature. This can be seen from the changes of the intensity of the peak at 1510–1513 cm^−1^ (CH=CH of the BM rings stretching vibrations, υCH=CH)); see Figure 3. It is clear that the intensity of this peak for both TG1 and IMG1 decreases at higher annealing temperature as expected when the cross-linked adduct is formed (Scheme 1).

**Figure 4:**
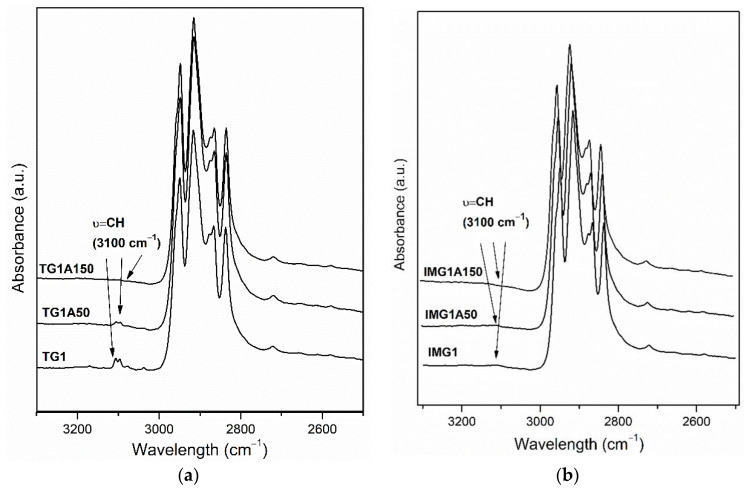
FTIR spectra of TG1 and its annealing products (**a**); and IMG1 and its annealing products (**b**) in the absorption band of 2500−3300 cm^−1^.

In addition to the peak at 1510–1513 cm^−1^, the peak at 3100 cm^−1^ which corresponds to =CH stretching vibrations of BM rings (υ=CH), also decreases at a higher annealing temperature [46,52]. As shown in Figure 4a, the peak at 3100 cm^−1^ for TG1 disappears after annealing at 150 °C, suggesting an increase of the cycloadduct formation at higher temperature. The peak at 3100 cm^−1^ is already absent in IMG1 (before the annealing) (Figure 4b). This suggests that there is a clear difference in the reactivity between imidazole and thiophene. It seems that the cross-linking reaction between imidazole and BM is faster compared with the one of thiophene and BM.

**Figure 5:**
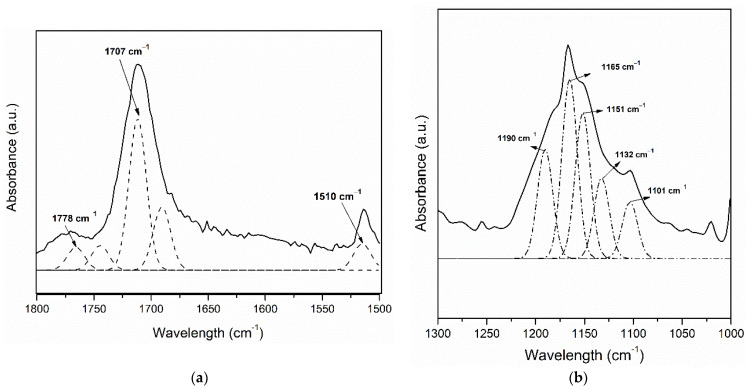
An example of deconvoluted spectrum of TG1A150 (ashed lines) within the wavelength of 1500−1800 cm^−1^ (**a**) and 1000−1300 cm^−1^ (**b**).

The quantification of these changes was made based on the deconvolution of the spectra from all products. An example of deconvoluted spectra of two different wavelengths: 1500–1800 cm^−1^ and 1000–1300 cm^−1^ of TG1A150 as shown in Figure 5.

The intensity of relevant peaks, which are the peak of cross-linked adduct (DA or Michael addition at 1186 cm^−1^) and the peak of CH=CH of the BM rings stretching vibrations (1510 cm^−1^), is normalized to the intensity of the absorption peak at 1707 cm^−1^ (C=O symmetric stretching, υCOsym). This peak is chosen since its intensity remains constant during the whole reaction sequence [53,54]. The intensity ratio of the peak at 1186 cm^−1^ (I_1184/1707_) and at 1510 cm^−1^ (I_1510/1707_) for TG1, IMG1 and their annealed products are given in Table 3.

The changes in the intensity after annealing are evident for all products (Table 3). It is obvious that the DA adduct is absent in TG1 (I_1184/1707_ = 0) and formed at higher temperature (TG1A50 and TG1A150). The intensity of the peak at 1186 cm^−1^ (I_1186/1707_) increases with temperature, while, on the contrary, the intensity of peak at 1510 (I_1510/1707_) decreases from 0.284 to 0.164 at higher annealing temperature. This signifies a higher degree of cross-linking of the TG1 product at higher annealing temperature.

In contrast with TG1, the cross-linking in IMG1 product is already formed even before annealing as indicated with I_1186/1707_ value of 0.153 (Table 3). This is in agreement with the observation of the peak at an absorption band of 3100 cm^−1^ (see above).

Evidently, imidazole is far more reactive compared with thiophene in the cross-linked reaction with BM. If the cross-linking of imidazole and BM proceeds via DA reaction (Scheme 1b), the higher reactivity of imidazole compared with thiophene in DA reaction is not surprising since it has a lower resonance energy (59 kJ/mol, less aromatic) compared with thiophene (121–129 kJ/mol) and thus imidazole has a higher tendency to react as a conjugated diene [55,56,57,58].

Thiophene shows less reactivity than imidazole; however, as in line with literature [28], it should be noted that DA reaction between thiophene and BM can proceed in a relatively mild condition (P = 0.1 MPa, *T* = 50 °C) compared with the other system involving thiophene and maleic anhydride as dienophile (P = 0.8 GPa, *T* = 100 °C) [59]. This shows a potential use of thiophene and BM as a pair of diene and dienophile in the DA chemistry for other polymer products as well.

**Figure 6:**
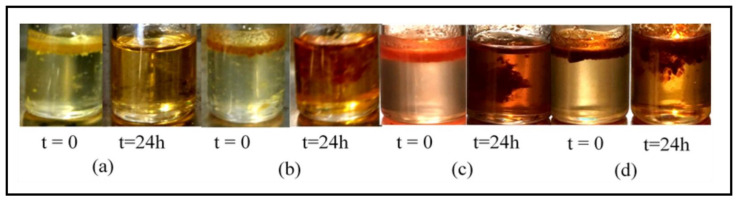
Solubility of TG1 (**a**), TG1A150 (**b**), IMG1 (**c**) and IMG1A150 (**d**) in dichlorobenzene at 120 °C for 24 h.

The difference in the degree of cross-linking of the product is also indicated by the difference in the solubility behavior of the TG1, IMG1 and their annealing products (Figure 6). TG1, which is not cross-linked (Table 3), is soluble in dichlorobenzene (DCB, Figure 6a). The product is cross-linked and became insoluble after annealing at 150 °C (Figure 6b). Moreover, as expected, IMG1 and IMG1A150 are not soluble, due to the presence of the cross-linking network in the product (Table 3).

The melt flow of the starting material (PPgMA) and the cross-linked product were measured with MVR instruments at a temperature range of 230–170 °C and load of 2.16 kg. The results are given in Table 4. It is obvious that a very high melt flow was observed for PPgMA. The melt flow value decreases after cross-linking as clearly shown for TG1A150 and IMG1A150. The decrease in melt flow value confirms the presence of a cross-linking network in the sample [60]. Moreover, the difference in melt flow value among the cross-linked product was also observed in the results (Table 4). TG1A150 has a very high flow at temperature of 230 °C while IMG1A150 has a lower melt flow (160 cm^3^/10 min) when measured at the same temperature (230 °C). This indicates the higher degree of cross-linking of IMG1A150 compared with TG1A150.

### 3.3. Rheological Behavior of the Cross-Linked Products

**Figure 7:**
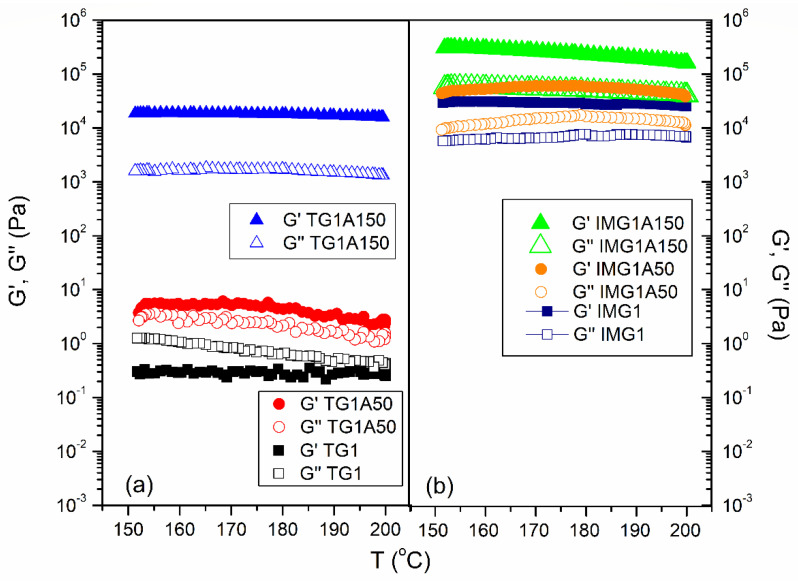
The change of storage (G′) and loss (G″) modulus of (**a**) TG1, TG1A50, TG1A150 and (**b**) IMG1, IMG1A50, IMG1A150 at different temperatures.

Figure 7a show the storage (G′ and loss (G″ modulus of TG1 and its annealed products measured at different temperatures (150–200 °C). The storage modulus (G′) of TG1 is somewhat lower than its loss modulus (G″, G″ > G′), indicating that there is no cross-linking and thus no network can be detected in the product [22]. However, the difference in the two modulus (G′ and G″) values decreases with temperature and the cross-over point is nearly reached at temperature of 200 °C. In this case, TG1 may be cross-linked at temperature higher than 200 °C (*T* > 200 °C), however limited to its degradation temperature.

In contrast with TG1, the G′ value of the annealed products (TG1A50 and TG1A150) is always higher than G″ value [22]. This confirms the presence of the cross-linked network in TG1A50 and TG1A150 irrespective of the temperature used in the rheology measurement. As shown in Figure 7a, the G′ value of TG1A50 decreases with temperature suggesting a lower degree of cross-linking at temperature higher than 150 °C. In this case, TG1A50 may be de-cross-linked at temperatures higher than 150 °C (*T* > 150 °C); however, only partial de-cross-linking takes place via retro DA reaction. The latter may be related to the possible aromatization process of the DA adduct at high temperatures (*T* > 150 °C) resulting in a more thermostable end product [61].

In other hand, a relatively constant G′ and G″ values at higher temperature is observed in the rheogram of TG1A150 (Figure 7a). This implies that no de-cross-linking appears in the product within the measurement windows. It seems that after annealing at 150 °C for 24 h, the TG1A150 product shows even higher thermostability compared with TG1A50. It is also important to note that the difference between G′ and G″ values of TG1A150 compared with TG1A50 (Figure 7a) increases. This may be strongly related to the higher degree of cross-linking formed at a higher annealing temperature.

The temperature sweep rheograms of IMG1 and its annealed products are shown in Figure 7b. In all cases, G′ values of IMG1 and its annealed products are higher than G″ (G′> G″) as expected for the presence of cross-linking in the product. The cross-linking degree of IMG1 and its annealed products are significantly higher compared with the cross-linked products based on thiophene (TG1, TG1A50, TG1A150). This is in agreement with the |η*| (complex viscosity) values obtained from frequency sweep measurements (see Figure S1, Supporting Information). The difference in the |η*| value is strongly related with the diversity in the cross-linking degree of the products [1,62]. In this aspect, a higher degree of cross-linking causes a more restricted chain mobility of the product and eventually leads to the higher (|η*|) value. Therefore, based on these results (Figure 7 and Figure S1), it is reasonable to conclude that IMG1A150 has the highest degree of cross-linking compared with the other products.

**Figure 8:**
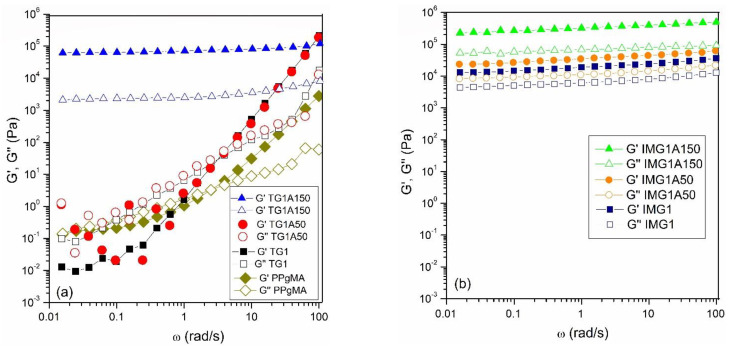
The change in storage (G′) and loss (G″) modulus of (**a**) PPgMA, TG1 and its annealed products and (**b**) IMG1 and its annealed products at various angular frequency (ω, rad/s) and measured at temperature of 160 °C.

Evidently, IMG1 already shows a relatively higher degree of cross-linking prior to annealing, while this is not the case for TG1 (Figure 7a,b) and for FG1 (furan based cross-linked products) as in agreement with the FT-IR and solubility measurements (TG1 and FG1 is still soluble in DCB). Detailed experimental results on cross-linking of PP with furan and bismaleimide were already reported in our recent publication [27]. The difference in reactivity of IMG1 compared with TG1 and FG1 prior to annealing suggests that the cross-linking between imidazole and bismaleimide may take place via a different reaction route than thiophene or furan with bismaleimide [27]. It is reasonable to assume that cross-linking of imidazole and bismaleimide proceeds via Michael addition (Scheme 1b) instead of Diels–Alder (Scheme 1c) reaction. Furthermore, if the reaction proceeds via DA, then one could expect the presence of a reversible cross-linked network in the TG1 and its annealed products. In this case, only an irreversible cross-linked network was present in the IMG1 and its annealed products, which is in line with the observations shown by Figure 7b and Figure 8.

The melt rheological properties of all the products were studied with frequency sweep measurements at temperature of 160 °C. Figure 8 shows the G′ and G″ values of the starting material (PPgMA) and its cross-linked products at various angular frequency (ω). In the starting material, a cross-over point appears at an angular frequency of 10 rad/s, separating the region between the liquid like (G″ > G′) melt and solid like (G′ > G″) melt behavior [63]. The cross-over point is still present in TG1 and TG1A50 and disappears in TG1A150 (Figure 8a). In the latter, the G′ value is always higher than G″ value and the products have a solid like melt behavior. This implies that both TG1 and TG1A50 have a reversible cross-linking network in their polymer structure while it is not the case for TG1A150. On the other hand, all IMG1 and its annealed products show no crossover point and exhibit only a solid like melt behavior (G′ > G″, Figure 8b). The absence of a cross-over point prior to annealing (IMG1 sample, see above) and its annealed products (IMG1A50 and IMG1A150) indicates an irreversibly cross-linked network in all imidazole products and eventually confirms that a cross-linking reaction of grafted imidazole with BM takes place via the irreversible Michael addition reaction (Scheme 1c).

**Figure 9:**
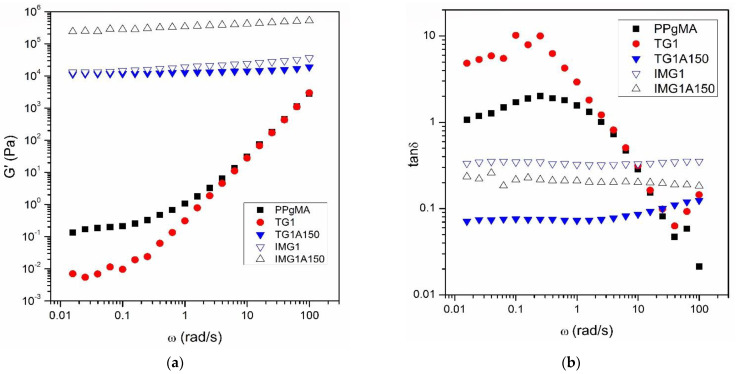
The change in storage modulus (G′) (**a**) and loss tan (tan δ) (**b**) of the cross-linked products at various angular frequency (ω, rad/s) and measured at temperature of 160 °C.

The observations made in Figure 8 also imply that the changes from liquid like (G″ > G′) to solid like (G′ > G″) melt behavior depend on the degree of cross-linking in these products. Thus, it also reasonable to assume that the product with the higher degree of cross-linking will have a higher storage modulus (G′) value and higher melt elasticity (Figure 9a) [62,64,65]. This argument agrees well with the result, since IMG1A150 with the highest degree of cross-linking has the highest melt elasticity value (Figure 9a).

Figure 9b shows the loss tangent (tan δ) values for PPgMA and its derivatives at different angular frequency (ω). It is obvious that, at a low frequency region, the tan δ value of the modified PPgMA products has a lower value compared with PPgMA except for TG1. These results suggest that, in the low frequency region, most of the cross-linked products have a higher melt strength compared with the starting material. The fact that TG1 has the lowest melt strength among other is due to the absence of the cross-linking in the product (see Figure 7a). Interestingly, the highest melt strength is not achieved by the product with the highest melt elasticity (highest G′ value) as commonly observed in the literature [63]. In our case, TG1A150 has the highest melt strength compared with IMG1A150 (see Figure 9b) although IMG1A150 has higher G′ value compared with TG1A150. This implies that the melt strength is not solely determined by the storage (G′) or loss modulus (G″) but also the interplay between the two parameters.

**Figure 10:**
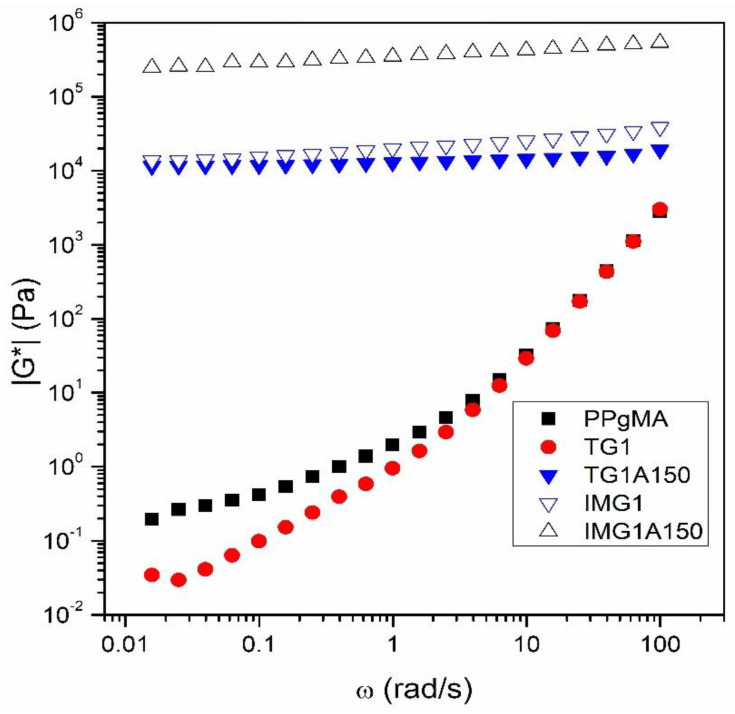
The change in complex modulus (|G*|) of the cross-linked products at various angular frequency (ω, rad/s) and measured at temperature of 160 °C.

Instead of the melt elasticity and strength, a difference in the product rigidity was also observed (Figure 10). Apparently, IMG1 and IMG1A150 show higher rigidity (|G*|) compared with TG1, TG1A150 and PPgMA [22]. The highest rigidity is accessible by IMG1A150. 

This finding indicates that the mechanical robustness of the final products may also be tuned by different reagents and different annealing temperatures employed in the DA reaction.

### 3.4. Thermal Properties of the Cross-Linked Products

The changes in the melting and crystallization behaviors of the maleated PP (PPgMA) and the cross-linked product were measured using DSC. The results are given in Table 5.

Clearly, the melting temperature (*T*_m_) of the PPgMA (*T* = 155 °C) decreases after cross-linking to a temperature of 149 °C (for TG1A150) and even lower *T*_m_ is achieved by IMG1A150 (146 °C). A slightly different trend was observed for the crystallization temperature (*T*_c_), melting enthalpy (Δ*H*_m_) and crystallization enthalpy (Δ*H*_c_) values. When comparing *T*_c_, Δ*H*_m_ and ΔH_c_ values of PPgMA and TG1, a higher value was observed. While it is not the case for IMG1, a lower Tc, Δ*H*_m_ and Δ*H*_c_ values compared with the ones in PPgMA were obtained (Table 5). These observations suggest that the presence of thiophene gives a positive influence compared with imidazole towards the nucleation density and eventually enhances the crystallization in the product [60].

Furthermore, the *T*_c_, Δ*H*_m_ and Δ*H*_c_ values for both TG1 and IMG1 decrease after annealing in line with the trend in *T*_m_ value. Obviously, the presence of the cross-linked network in the maleated PP after DA reaction lead to reduction of melting and crystallization properties including the degree of crystallinity of the products. These results can be explained by the fact that the formation of the cross- linked network may create defects in the crystallization structure, resulting in the retardation of crystallization and a lower crystallization rate [66]. Eventually, this leads to a decrease in crystallinity of the cross-linked products.

**Figure 11:**
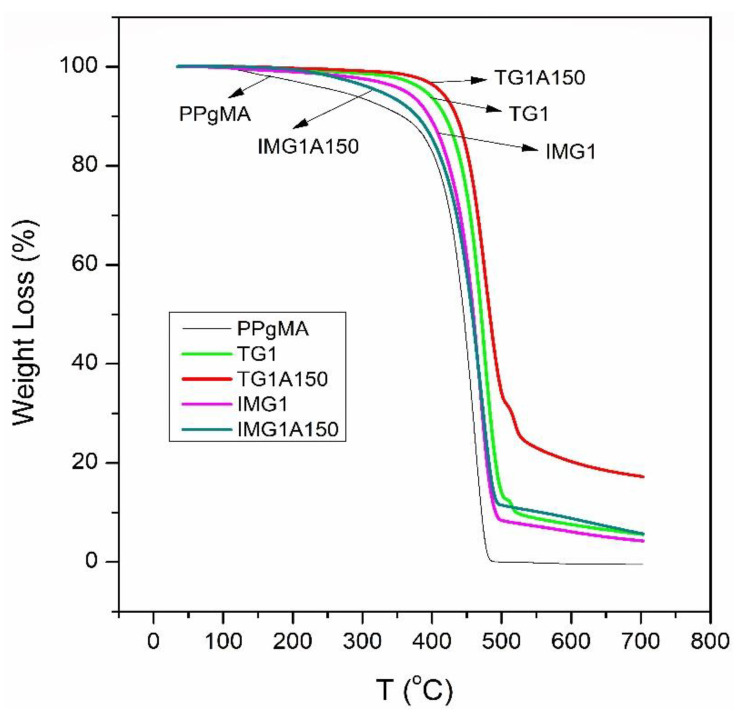
Thermal stability of maleated PP and its cross-linked products.

In addition to the changes of the melting and crystallization properties, the thermal stability of the cross-linked product (TG1, TG1A150, IMG1, IMG1A150) also differs significantly compared with the starting material (PPgMA), see Figure 11. It is evident that all of the cross-linked products have a higher degradation temperature compared with PPgMA (Figure 11). This suggests that the presence of cross-linking in the polymer backbone has successfully improved the thermal stability of the final product [13].

As shown in Figure 11, the highest thermal stability was obtained by TG1A150 (*T*_degradation_ = 340–350 °C) while PPgMA starts to degrade already at temperature of 106–110 °C. When comparing with the thiophene cross-linked products (TG1 and TG1A150), it is obvious that the imidazole cross- linked products (IMG1 and IMG1A150) have a lower thermal stability though the products have a higher degree of cross-linking. The degradation temperature of IMG1 starts at 270–280 °C while IMG1A150 starts to degrade even at lower temperatures (235–245 °C). The less thermal stability of imidazole cross-linked products compared with thiophene may be the result of the higher disruption in the crystalline structure and the lower crystallinity in the imidazole cross- linked products as confirmed with DSC analysis (Table 5) [67].

### 3.5. Reversibility of the Cross-Linked Products

It is also interesting to evaluate the thermal reversibility of the cross-linked products which should be possible due to the retro DA reaction, especially for thiophene based cross-linked products (Scheme 1a). In this work, the thermal influence on the cross-linking reaction is evaluated at two different annealing temperatures which are temperatures of 50 °C and 150 °C. It is clear that, after annealing at a temperature of 150 °C, a higher cross-linking degree in the product was obtained for both thiophene and imidazole cross-linked products as indicated by an increase in the I_1184/I1707_ (FT-IR results, Table 2), the insolubility of the cross-linked products (Figure 6) and an increase in the complex viscosity value (rheology measurement, Figure S1). The rheology measurement using a temperature sweep program shows that, even at higher temperatures than 150 °C (*T* > 150 °C), only partial de-cross-linking was observed in TG1A50 until a temperature of 200 °C (Figure 7), while the other annealed products (TG1A150 and all IMG1 cross-linked products) show no de-cross-linking in the rheograms. In addition, for TG1, it seems that the DA cross-linking is possible to proceed at a temperature higher than 200 °C (Figure 7).

Based on these observations so far, it is plausible that, within the experimental window, temperature has a positive effect on the cross-linking, in this case affecting mainly the equilibrium reaction (Scheme 1a) towards the DA reaction for thiophene and increases Michael addition reaction rates for imidazole (Scheme 1c). Moreover, another important finding is obtained by MVR (Table 3). The results suggest that not only do the cross-linked products show sufficient flow, but also the products are melt processable during the MVR measurement. Surprisingly, the latter also happens on IMG1 and IMG1A150 products, which shows no reversible network in their structure (rheology measurements, Figure 7b and Figure 8b). This indicates that the reversibility in the thiophene/imidazole cross-linked product is not solely determined by temperature but also the possible interplay between temperature and shear stresses. However, further studies are still required in the future to elucidate the role of both temperature and shear stresses on the reversibility of the novel products.

## 4. Conclusions

This work demonstrates the successful application of 2-thiophenemethyl amine (TMA),1-(3 aminopropyl) imidazole (API)) and 1,1′-(methylenedi-4,1-phenylene) bismaleimide (BM)as the cross-linking agents in the synthesis of cross- linked PP using maleated PP (PPgMA). The cross-linking of thiophene and bismaleimide reaction proceeds via Diels–Alder (DA) that involves two consecutive reaction steps, i.e., the grafting of TMA onto maleated PP and the DA reaction of thiophene pendant group with BM. On the other hand, the cross-linking of imidazole and bismaleimide takes place via the Michael addition reaction pathway, which also consists of two consecutive reaction steps, i.e., the grafting of API onto maleated PP and the Michael addition reaction of imidazole pendant group with BM.

The difference in reactivity between the two reagents (TMA and API) was observed in each reaction step. API gives a higher grafting efficiency (% grafted = 65%) compared with TMA (% grafted = 29.8%). Moreover, the imidazole pendant group gives higher reactivity in the cross-linking reaction as indicated by the presence of the cross-linking adduct (CH–N aliphatic stretching, υCH–N, observed at an absorption band of 1186 cm^−1^) after the reaction (IMG1), while, in contrast, prior to annealing, no cycloadduct was observed in the thiophene product (TG1). After annealing, the degree of cross-linking in the products increases and no de-cross-linking was observed in several products (TG1A150, IMG1A50 and IMG1A150).

Not only the chemistry between DA and Michael addition, but the difference in rheological and thermal properties was also observed between the imidazole and thiophene cross-linked products. Even though the imidazole cross-linked products have better melt properties (higher melt strength, rigidity and complex viscosity), the imidazole product shows less thermal stability compared with thiophene counterparts as probably caused by the lower crystallinity of the product after the cross-linking. Moreover, both imidazole and thiophene cross-linked products show significantly higher melt properties and thermal stability compared with the starting material. The superior product properties and the fact that the cross-linked product is still melt processable may give a new perspective on the synthesis of the cross-linked PP via DA and Michael addition chemistry and the application of the product on the industrial scale.

## Figures and Tables

**Table 1:** Detailed experimental condition of grafting thiophene and imidazole and subsequent cross-linking with BM.

Reaction Steps	Polymer	Product Code	BM Intake (Mol Equiv)	Reagent Intake (Mol Equiv)	Temperature (°C)	Reaction Time
A. Grafting with: TMA	PPgMA	TG0		4	160	10 min
API	PPgMA	IMG0		4	160	10 min
B. Cross-linking with BM						
Prior Annealing	TG0	TG1	0.5		50	180 min
	IMG0	IMG1	0.5		50	180 min
After Annealing	TG1	TG1A50			50	24 h
		TG1A150			150	24 h
	IMG1	IMG1A50			50	24 h
		IMG1A150			150	24 h

**Table 2:** Elemental analysis on maleated PP and the grafted products.

						MA		
	N%	C%	H%	S%	O% ^b^	% mol	% wt.	% Grafted ^a^
PPgMA	0	81.93	13.70	-	4.37	0.091	8.9	
TG0	0.38	83.92	13.88	1.14	0.685			29.8
IMG0	2.49	82.06	13.31	-	2.15			65

^a^ % grafted = mol N in the grafted products/mol MA in maleated PP. ^b^ The remaining composition is assumed to consist oxygen.

**Table 3:** Intensity ratio of relevant cross-linking peaks of thiophene and imidazole with bismaleimide.

	I_1510/1707_	I_1186/1707_
TG1	0.289	0
TG1A50	0.252	0.099
TG1A150	0.164	0.296
IMG1	0.329	0.153
IMG1A50	0.345	0.195
IMG1A150	0.233	0.293

**Table 4:** Melt Volume Rate of PPgMA and its cross-linked products.

No	Sample	Melt Volume Rate (cm^3^/10 min)	
		*T* = 170 °C	*T* = 230 °C
1	PPgMA	Very high flow	Very high flow
2	TG1	Very high flow	Very high flow
3	TG1A150	79	Very high flow
4	IMG1	Very high flow	Very high flow
5	IMG1A150	n.m. *	160

* n.m.: not measured.

**Table 5:** Melting and crystallization properties of thiophene bismaleimide products.

	*T*_m_ (°C)	*T*_c_ (°C)	Δ*H*_m_ (J/g)	Δ*H*_c_ (J/g)
PPgMA	155	108	63.4	71.6
TG1	151	121	76.3	77.6
TG1A150	149	114	63.5	69.6
IMG1	148	121	50.7	60.3
IMG1A150	146	117	29.9	44.2

## Data Availability

Data from this study are available upon request from the corresponding author.

## Supplementary Materials

The following supporting information can be downloaded at: https://www.mdpi.com/article/10.3390/polym14112198/s1, Figure S1: The change in complex viscosity (|η*|) of the cross-linked products at various angular frequency (ω, rad/s) and measured at temperature of 160 °C.
