# Supplementary material for: Cross-Linking of Polypropylene with Thiophene and Imidazole
Source: Polymers (Basel). 2022 May 28;14(11):2198. doi: 10.3390/polym14112198 (PMC9182647; doi:10.3390/polym14112198)
Supplement: Supplementary file 1 [file polymers-14-02198-s001.zip › polymers-1717057-supplementary.pdf]

Supporting Information

Article

Cross-Linking of Polypropylene with Thiophene and Imidazole

Henky Muljana [1,2,*], Klaas Remerie [3], Gert Boven [3], Francesco Picchioni [2] and Ranjita K. Bose [2,*]

1 Department of Chemical Engineering, Parahyangan Catholic University, Ciumbuleuit 94, Bandung 40141, Indonesia
2 Department of Chemical Engineering, ENTEG, University of Groningen, Nijenborgh 4, 9747 AG Groningen, The Netherlands; f.picchioni@rug.nl
3 SABIC, Plasticslaan 1, P.O. Box 117, 4600 AC Bergen op Zoom, The Netherlands; k.remerie@home.nl (K.R.); gert.boven@sabic.com (G.B.)
* Correspondence: henky@unpar.ac.id (H.M.); r.k.bose@rug.nl (R.K.B.)

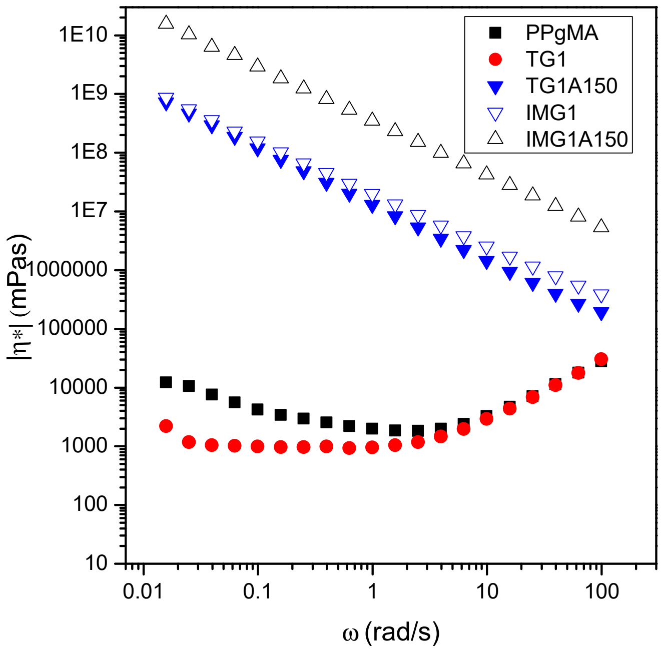

Figure S1. The change in complex viscosity (|η*|) of the cross-linked products at various angular frequency (ω, rad/s) and measured at temperature of 160 °C.
